# Editorial: Clinical guidelines in schizophrenia: applications and evaluation

**DOI:** 10.3389/fpsyt.2023.1261696

**Published:** 2023-08-22

**Authors:** Shinsuke Hidese, Signe Düring, Rafael Penadés

**Affiliations:** ^1^Department of Psychiatry, School of Medicine, Teikyo University, Tokyo, Japan; ^2^Competency Center for Dual Diagnosis, Copenhagen University Hospital–Mental Health Services Copenhagen, Copenhagen, Denmark; ^3^Hospital Clinic of Barcelona, University of Barcelona, Biomedical Research Networking Center for Mental Health Network, Barcelona, Spain

**Keywords:** clinical guideline, negative symptom, psychometric tool, schizophrenia, violence

The present Research Topic aimed to deal with the latest research focused on the reliability or validity of clinical guidelines in the evaluation of schizophrenia. In addition, cultural, socioeconomic, or other factors relating to the application of clinical guidelines in schizophrenia were objectives of this Research Topic. Although it was not initially intended, the submission content has consequently become about negative symptoms in schizophrenia and related assessment and treatment guidelines. Therefore, the title of the Research Topic could be rewritten as “*Clinical guidelines in schizophrenia: applications and evaluation focused on negative symptoms*.”

The Clinical Assessment Interview for Negative Symptoms (CAINS) is an instrument for the measurement of negative symptoms, designed to address the five domains (i.e., blunted affect, alogia, asociality, anhedonia, and avolition) (1). Laraki, Bayard et al. confirmed the validity and reliability of a French version of the CAINS in 84 outpatients with schizophrenia; the French CAINS can be useful in clinical and research settings. Zhou et al. determined the optimal cutoff scores for a total score of 40 and its subscales (i.e., communication, emotion, and motivation factors) in the Chinese version of a 15-item negative symptom assessment to identify prominent negative symptoms in 199 patients with schizophrenia. These validation studies are expected to provide evidence for respective psychometric tools to assess the severity of negative symptoms in schizophrenia in each country.

Among the negative symptoms, Laraki, Lebrun et al. reported that anhedonia, measured using the composite score of the Anhedonia/Asociality subscale of the Scale for the Assessment of Negative Symptoms, is positively associated with the Fatigue Impact Scale, a social role scale, even when controlling for depression in 51 French patients with schizophrenia. Although the relationship between negative symptoms and violence in schizophrenia is still unclear, Guo et al. showed that the History of Violence, Clinical Risk Assessment Scale factors young age at first violent incident (H2), impulsivity (H4), and relationship instability (C2) as well as the Psychopathy Checklist-Revised antisocial factor score are risks of violence history in 507 male patients with schizophrenia in China. These symptomatic studies have revealed, at least in part, the clinical variables related to anhedonia and violence in schizophrenia using corresponded psychometric tools.

European Psychiatric Association guidance states that treatment including pharmacological, exercise, and psychosocial interventions has a lack of advanced evidence and remains inappropriate for formulating recommendations for primary, persistent, or predominant negative symptoms in schizophrenia (2). Furthermore, a review of schizophrenic negative symptoms in a large geographic region reported no obvious differences in the treatment of negative symptoms among published guidelines (3). These limitations warrant further studies to bridge the gap between existing guidelines and dimension-specific multi-level framework pathophysiological mechanisms of negative symptoms mapped on at least two dimensions (i.e., diminished expression and apathy), especially for the development of biological, psychosocial, and combined treatment approaches (4). Moreover, co-cited worldwide reference networks suggest that the conceptualization and treatment of negative symptoms are preferentially based on specific field of research trends such as evidence synthesis, non-pharmacological treatments, and computational psychiatry in the future (5).

## Author contributions

SH: Writing—original draft. SD: Writing—review and editing. RP: Writing—review and editing.
